# Effect of COVID-19 Vaccines on Reducing the Risk of Long COVID in the Real World: A Systematic Review and Meta-Analysis

**DOI:** 10.3390/ijerph191912422

**Published:** 2022-09-29

**Authors:** Peng Gao, Jue Liu, Min Liu

**Affiliations:** 1 Department of Epidemiology and Biostatistics, School of Public Health, Peking University, Xueyuan Road No. 38, Haidian District, Beijing 100191, China; 2 Institute for Global Health and Development, Peking University, Yiheyuan Road No. 5, Haidian District, Beijing 100871, China

**Keywords:** COVID-19 vaccine, long COVID, post COVID-19 condition, systematic review, meta-analysis

## Abstract

The coronavirus disease 2019 (COVID-19) is still in a global pandemic state. Some studies have reported that COVID-19 vaccines had a protective effect against long COVID. However, the conclusions of the studies on the effect of COVID-19 vaccines on long COVID were not consistent. This study aimed to systematically review relevant studies in the real world, and performed a meta-analysis to explore the relationship between vaccination and long COVID. We systematically searched PubMed, Embase, Web of science, and ScienceDirect from inception to 19 September 2022. The PICO (P: patients; I: intervention; C: comparison; O: outcome) was as follows: patients diagnosed with COVID-19 (P); vaccination with COVID-19 vaccines (I); the patients were divided into vaccinated and unvaccinated groups (C); the outcomes were the occurrence of long COVID, as well as the various symptoms of long COVID (O). A fixed-effect model and random-effects model were chosen based on the heterogeneity between studies in order to pool the effect value. The results showed that the vaccinated group had a 29% lower risk of developing long COVID compared with the unvaccinated group (RR = 0.71, 95% CI: 0.58–0.87, *p* < 0.01). Compared with patients who were not vaccinated, vaccination showed its protective effect in patients vaccinated with two doses (RR = 0.83, 95% CI: 0.74–0.94, *p* < 0.01), but not one dose (RR = 0.83, 95% CI: 0.65–1.07, *p* = 0.14). In addition, vaccination was effective against long COVD in patients either vaccinated before SARS-CoV-2 infection/COVID-19 (RR = 0.82, 95% CI: 0.74–0.91, *p* < 0.01) or vaccinated after SARS-CoV-2 infection/COVID-19 (RR = 0.83, 95% CI: 0.74–0.92, *p* < 0.01). For long COVID symptoms, vaccination reduced the risk of cognitive dysfunction/symptoms, kidney diseases/problems, myalgia, and sleeping disorders/problems sleeping. Our study shows that COVID-19 vaccines had an effect on reducing the risk of long COVID in patients vaccinated before or after SARS-CoV-2 infection/COVID-19. We suggest that the vaccination rate should be improved as soon as possible, especially for a complete vaccination course. There should be more studies to explore the basic mechanisms of the protective effect of COVID-19 vaccines on long COVID in the future.

## 1. Introduction

The coronavirus disease 2019 (COVID-19) caused by severe acute respiratory syndrome coronavirus 2 (SARS-CoV-2) infection is still in a global pandemic state. According to the World Health Organization (WHO), there have been 599 million confirmed cases of COVID-19 as of 31 August 2022 [1]. Although patients recover from acute symptoms of COVID-19, it is worrying that certain studies have pointed out that sequelae in some patients (adults and children) may last weeks or even months [2,3,4]. However, COVID-19 might have detrimental sequelae even after the post-acute phase, depicting a new pathological condition—“post-COVID-19 syndrome (PCS)” or “long COVID” [5]. Long COVID is also known as the post-COVID-19 condition, long-term symptoms following SARS-CoV-2 infection, post-acute COVID-19 syndrome, or post-acute sequelae of SARS-CoV-2 infection, etc. [6,7]. The clinical case definition of long COVID, published by the WHO, is that it occurs in individuals with a history of probable or confirmed SARS-CoV-2 infection, usually 3 months from the onset of COVID-19 with symptoms, and lasts for at least 2 months, and it cannot be explained by an alternative diagnosis [8]. Long COVID affects multiple organs, and common symptoms include tiredness/fatigue, dyspnea/difficulty breathing, cough, chest pain, diarrhea, headache, impaired balance and gait, insomnia, joint pain, myalgia and weakness, neurocognitive issues, palpitations, pins and needles, rash, and hair loss [9,10].

According to the WHO, a total 12 billion COVID-19 vaccines have been administered as of 23 August 2022 [1]. COVID-19 vaccines could prevent SARS-CoV-2 infection, symptomatic COVID-19, and severe COVID-19, although their effectiveness was found to decline as time went by [11]. COVID-19 vaccines also had a good effectiveness against COVID-19-related hospitalization, admission to the intensive care unit, and death in a real-world setting [12]. The protective effect of COVID-19 vaccines has also been observed among children [13]. However, it is not clear that whether COVID-19 vaccines can prevent long COVID [6]. A cohort study in healthcare personnel with confirmed COVID-19 showed that the prevalence of reporting one or more COVID-like symptom 6 weeks after the onset of illness in the vaccinated group was lower compared with the unvaccinated group [14]. Another study reported that the number of vaccine doses was associated with lower long COVID incidence among healthcare workers who had not required hospitalization [15]. However, one study indicated that the mean number of post-acute sequelae of COVID-19 (PASC) symptoms reported each month during the follow-up period and the odds of full recovery from PASC were comparable between vaccinated and unvaccinated groups [16].

As far as we know, at present, only one preprint [17] and one published article [18] have provided systematic reviews on this topic without a meta-analysis. Therefore, we conducted this systematic review with a meta-analysis to quantitatively explore the effect of COVID-19 vaccines on long COVID, and to provide scientific evidence and suggestions. The PICO (P: patients; I: intervention; C: comparison; O: outcome) was as follows: patients diagnosed as having COVID-19 (P); vaccination with COVID-19 vaccines (I); the patients were divided into vaccinated and unvaccinated groups (C); the outcomes were the occurrence of long COVID, as well as the various symptoms of long COVID (O).

## 2. Materials and Methods

### 2.1. Registration and Search Strategy

Our study was registered in Prospective Register of Systematic Reviews (PROSPERO, ID: CRD42022340472). The study process followed the Preferred Reporting Items for Systematic Reviews and Meta-Analysis (PRISMA) guidelines strictly. We systematically searched PubMed, Embase, Web of science, and ScienceDirect from inception to 19 September 2022. One part of the search terms was “vaccine” and its synonyms, the other part of the search terms was “long COVID” and its synonyms. The two parts were logically connected by “AND”. The complete search strategy is shown in Table S1 of the Supplementary Materials. In addition, we checked the reference lists of relevant reviews for more studies.

### 2.2. Study Selection

In this study, no matter how the studies considered defined long COVID, they would be included if they met the inclusion criteria. The inclusion criteria were as follows: (1) studies in extracted data could be extracted on the number of long COVID patients for vaccinated and unvaccinated patients; (2) studies conducted on humans, not on animals or cells; and (3) cohort study design, case-control study, or cross-sectional study. The exclusion criteria were as follows: (1) being irrelevant to this study (animal experiments, basic medical research, using models to evaluate, or participants obviously were vaccinated after long COVID, etc.); (2) study design not needed (clinical trial, review, case series, case report, conference abstract, or comment); (3) data not available (data were unable to be extracted or unable to be used for quantitative synthesis); and (4) duplicate articles.

EndNote (version 20, Tomson ResearchSoft, Stanford, CA, USA) software was used to exclude duplicates and to manage the results obtained by the search. In order to obtain as much data as possible, during the screening by title and abstract, only studies that obviously met the exclusion criteria were excluded. The rest of the records were selected by reading the full texts. Then, the eligible articles that met the inclusion criteria were finally included. Study selection (as well as data extraction and quality assessment of the included studies below) was done independently by two researchers, and disagreements were resolved through discussion or through a decision by a third researcher.

### 2.3. Data Extraction

The following information and data of included studies were extracted: (1) basic information, namely first author, title, publication time, and study design; (2) characteristics of the population, namely nationality, age, sample size, and follow-up time; (3) information of vaccination, namely vaccination time, type of vaccine, and number of doses; and (4) information of outcomes, namely outcome, observation period, number of long COVID patients, and number of long COVID symptoms.

### 2.4. Quality Assessment of Included Studies

For cohort studies and case-control studies, the Newcastle Ottawa scale [19] (NOS) was used to evaluate the risk of bias. The results of NOS include a low risk of bias (7–9 scores), moderate risk of bias (4–7 scores), and high risk of bias (0–3 scores). For cross-sectional studies, the checklist recommended by Agency for Healthcare Research and Quality [20] (AHRQ) was used, and the results include low risk of bias (8–11 scores), moderate risk of bias (4–7 scores), and high risk of bias (0–3 scores).

### 2.5. Statistical Analysis

In this study, exposure was vaccination with COVID-19 vaccines. We divided the population into vaccinated and unvaccinated groups. Participants who received one or more doses of COVID-19 vaccines were considered to be in the vaccinated group. The outcomes were the occurrence of long COVID (having at least one symptom) and various symptoms of long COVID. The risk ratio (RR) was calculated to assess the risk of developing long COVID in the vaccinated group compared with the unvaccinated group. In addition, we performed a subgroup analysis by age (<60 or ≥60 years), number of vaccine doses (one dose or two doses), vaccination time (before SARS-CoV-2 infection/COVID-19 or after SARS-CoV-2 infection/COVID-19), and definition of long COVID (“presence of symptoms more than 4 weeks after SARS-CoV-2 infection/COVID-19 diagnosis” or “other definitions”). For the primary meta-analysis, a sensitivity analysis was performed to assess the robustness, and the Egger test was conducted to assess the publication bias.

We calculated I^2^ statistics to show the heterogeneity between studies. The model used to pool the effect value was chosen based on the heterogeneity. When I^2^ ≤ 50, this showed that the heterogeneity was low to moderate, and a fixed-effect model was used. When I^2^ > 50, this showed that the heterogeneity was moderate to high, and a random-effects model was used. Statistical analysis was done using Review manager (version 5.4.1, The Cochrane Collaboration, London, UK) and R (version 4.1.0, Robert Gentleman and Ross Ihaka, Auckland, New Zealand) software.

## 3. Results

### 3.1. Characteristics of Included Studies

**Figure 1:**
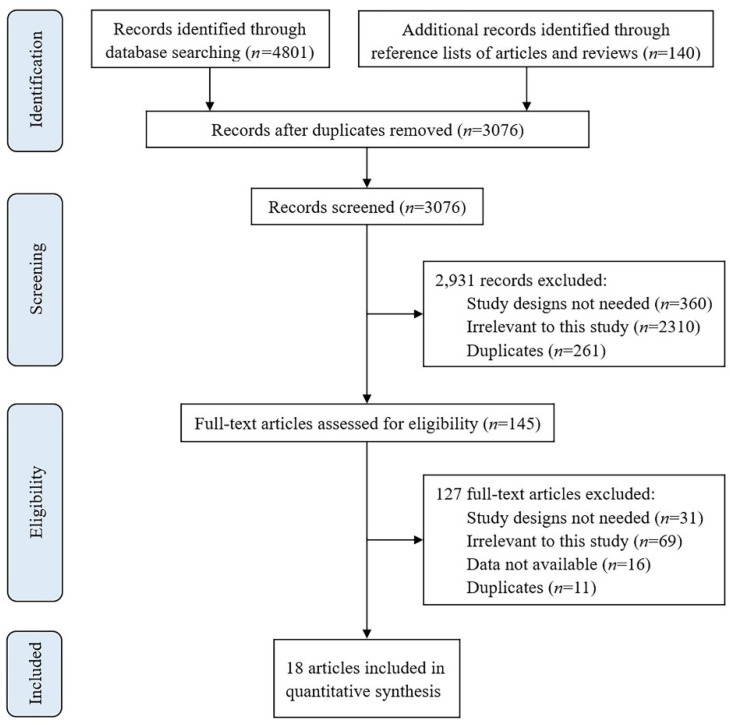
Flowchart of the study selection.

The process of study selection is shown in Figure 1. By searching the databases and checking the reference lists, we obtained 4941 records. A total of 3076 records were screened by reading titles and abstracts after duplicates were removed by the software. Finally, 18 eligible studies were included for quantitative synthesis after reading the full texts of 145 articles. The main information and the data for the meta-analysis of the included studies are shown in Table 1 and Table S2 of the Supplementary Material, respectively. Among them, 15 articles were accepted or published [15,16,21,22,23,24,25,26,27,28,29,30,31,32,33] and 3 articles were preprints [7,34,35]. All of the studies were observational, including 12 cohort studies, 1 case-control study, and 5 cross-sectional studies. Most of the populations were from the USA, UK, and Spain. There were more than 100,000 participants from each of these three countries. Three studies conducted in India, Switzerland, and Saudi Arabia each had a sample size over 1000. The other sample sizes were below 1000. The populations were mainly vaccinated with mRNA vaccines. Part of the populations were vaccinated after they had had SARS-CoV-2 infection or COVID-19. The definition of long COVID varied between studies. Only one study followed the definition published by the WHO (details of definition could be seen in the introduction). Three studies followed the definition published by the National institute for Health and Care Excellence (NICE) (details of the definition can be seen in the footer of Table 1). The definitions of five studies were similar to the NICE definition, because they used “more than 4 weeks” as the cut-off value for the observation time of long COVID-19 symptoms. Eight studies followed other definitions and two studies had no clear definition. In terms of quality assessment, only four studies had moderate risk of bias, and the rest had a low risk of bias. Overall, the quality of the included studies was good. The details of the quality assessment are shown in Table S3 of the Supplementary Material.

### 3.2. Primary Meta-Analysis and Sensitivity Analysis

**Figure 2:**
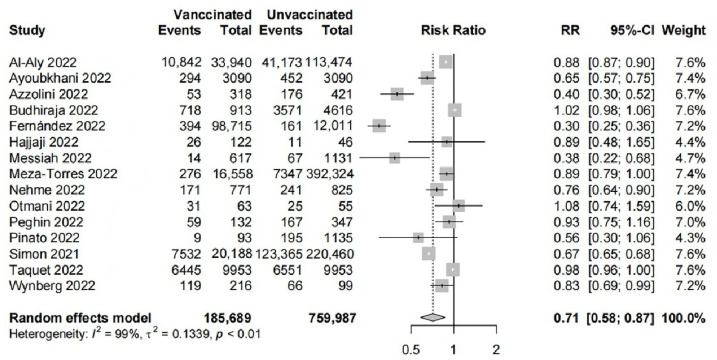
Forest plot of the effect of vaccination on long COVID [7,15,16,21,23,24,25,26,27,28,29,31,33,34,35].

**Figure 3:**
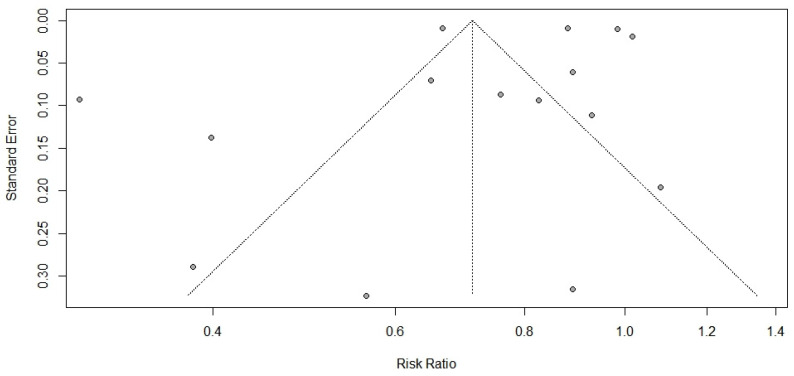
Funnel plot.

The results of the primary meta-analysis are shown in Figure 2. There was high heterogeneity between studies. Fifteen studies with 185,689 participants in the vaccinated group and 759,987 participants in the unvaccinated group were pooled using a random-effects model. RR = 0.71 (95% CI: 0.58–0.87, *p* < 0.01) indicated that the vaccinated group had a lower risk of developing long COVID compared with the unvaccinated group. The funnel plot is shown in Figure 3. The result of the Egger test (t = −0.46, df = 13, *p*-value = 0.65) suggested no publication bias in the primary meta-analysis.

All of the participants in the two studies (Pinato 2022 [29] and Hajjaji 2022 [31]) were patients with cancer, and we excluded this study for the sensitivity analysis. The pooled RR = 0.71 (95%CI: 0.57–0.88, *p* < 0.01) with a high heterogeneity (I^2^ = 99%) was almost the same as the primary meta-analysis. We excluded preprints (Ayoubkhani 2022 [7], Simon 2021 [34], and Budhiraja 2022 [35]) for the sensitivity analysis. The pooled RR = 0.70 (95%CI: 0.58–0.89, *p* < 0.01) with a high heterogeneity (I^2^ = 96%) still indicated that COVID-19 vaccines had a protective effect on long COVID. In addition, regardless of which study was excluded separately, the difference in the incidence of long COVID between two groups still was statistically significant. The RR value ranged from 0.69 to 0.77. More details of the sensitivity analysis are shown in Table S4 of the Supplementary Material.

### 3.3. Subgroup Analysis

The results of the subgroup analysis are shown in Table 2. For the number of doses, the protective effect of vaccination on long COVID was only found in the population vaccinated with two doses. For age, a protective effect was not found in either subgroup. For vaccination time, vaccination reduced the risk of developing long COVID in both the “before SARS-CoV-2 infection/COVID-19” subgroup and “after SARS-CoV-2 infection/COVID-19” subgroup. For definition, the results of the meta-analysis were both statistically significant.

### 3.4. Meta-Analysis for Long COVID Symptoms

The results of meta-analysis for long COVID symptoms are shown in Table 3. Compared with the unvaccinated group, the vaccinated group had a lower risk of cognitive dysfunction/symptoms, kidney diseases/problems, myalgia, and sleeping disorders/problems sleeping.

## 4. Discussion

As far as we know, our study is the first systematic review with a meta-analysis to assess the effect of COVID-19 vaccines on long COVID. A total 18 articles were included. In the primary meta-analysis, vaccination showed the effect of reducing the risk of long COVID. Considering the high heterogeneity between studies, the primary meta-analysis could be unstable, so we performed a sensitivity analysis. Each time a study was excluded for pooled RR evaluation, the protective effect of the vaccine always existed, indicating that the primary meta-analysis was stable. Because there are not many studies examining this topic, we included preprints. However, the preprints have not been peer-reviewed. We performed a sensitivity analysis by excluding three preprints. The result still supported the protective effect of COVID-19 vaccines. In the subgroup analysis, the results showed that people who received one dose vaccine did not acquire protection against long COVID, while those who received two doses did. Based on the data and the results of this paper, we cannot know the exact reason. We consider that it may be related to the higher vaccine effectiveness against symptomatic COVID-19 in people who received two doses of the vaccine. Several previous studies have confirmed this higher effectiveness [37,38,39]. Based on this, we recommend that people vaccinated with only one dose of COVID-19 vaccines should receive a second dose as soon as possible. Our study also found that the significance of vaccination is not limited to preventing SARS-CoV-2 infection and COVID-19. Vaccination in people who already have been infected with SARS-CoV-2 or COVID-19 is still effective at preventing long COVID. Based on this, we recommend that patients with SARS-CoV-2 infection or COVID-19 could choose to be vaccinated in order to prevent long COVID. The definition of long COVID was different between the included studies. Among the various definitions, more studies used “more than 4 weeks” as the observation period of long COVID symptoms. We divided these studies into one subgroup, and the articles that used other definitions into another subgroup. The results showed that the vaccine had a protective effect against long COVID in both subgroups. However, we believe that the difference in definitions brought an objective problem to our study. In future studies, we recommend that researchers use the NICE or WHO definitions in order to better describe what the outcome (long COVID) is in their articles, especially for review articles.

In this study, the protective effect of COVID-19 vaccines on long COVID symptoms could only be found in cognitive dysfunction/symptoms, kidney diseases/problems, myalgia, and sleeping disorders/problems sleeping. The pooled effect values of the other symptoms were negative. We believe this may be related to the small number of included studies. For most symptoms, only two or three studies were included for calculating the RRs, and most of the data were from three articles (Budhiraja 2022 [35]; Kuodi 2022 [32]; Taquet 2022 [24]). Not only that, in the study by Taquet, the number of patients with outcomes was high. This weight was very high (even higher than 90%) when the effect values were calculated, which had an impact on the results. The same situation with the small number of included studies also occurred in the subgroup analysis according to age. More original studies are needed to assess the effect of COVID-19 vaccines on these symptoms. In fact, for all of the outcomes, more studies should be included to obtain more reliable results.

The results of some previous studies support our study. One study indicated that COVID-19 vaccination reduced the likelihood of developing long COVID symptoms 12 weeks after infection, and found a sustained improvement over time in people who received two doses of the vaccine [40]. A reduction in the prevalence of one or more of the post-SARS-CoV-2 symptoms (difficulty concentrating or memory loss, fatigue, headache, loss of change in smell, loss of or change in taste, and shortness of breath) was significantly associated with the use of COVID-19 vaccines [21]. In people with SARS-CoV-2 infection, patients who were vaccinated had a significantly lower risk of developing 24 sequelae compared with patients who were not vaccinated [33]. After patients with COVID-19 were discharged from the hospital, persistent symptoms had an impact on their health and reduced their quality of life [41]. In a survey of 2550 people, 32% of participants were unable to live alone without assistance 6 months after onset, and the work of 75% of participants was affected after an average of 7 months into long COVID [42]. A study from Indonesia found that full vaccination improved the health-related quality of life among patients with COVID-19 6 months after hospital discharge, and suggested that COVID-19 survivors be vaccinated [43].

The pathophysiology of long COVID and the mechanism of effect of COVID-19 vaccines on long COVID are not very clear. Varying extents of organ damage, persistence of chronic inflammation, and immune response/auto antibody generation may be the causes of long COVID [9]. In patients with long COVID, persistently elevated inflammatory makers could be observed [44]. A study reported that SARS-CoV-2 damages the neurons, directly or indirectly, involving the central nervous system and the peripheral nervous system, leading to neurological sequelae [45]. Moreover, a hypothesis of persistent and occult virus presence has been proposed after the identification of viral particles in organs after acute infection [46]. Vaccination may decrease the risk of long COVID by increasing antibody titers and potentially eliminating viral reservoirs [47]. This may explain the result (in our subgroup analysis) that vaccination after SARS-CoV-2 infection/COVID-19 is still useful for preventing long COVID. Another mechanism is that vaccines can reduce the severity of acute SARS-CoV-2 infection, thus leading to a lower risk of developing organ or systemic derangements [18]. Severe COVID-19 in the acute phase during hospitalization increased the risk of long COVID [48,49]. This may explain the result (in our subgroup analysis) that vaccination before SARS-CoV-2 infection/COVID-19 has a protective effect on long COVID.

We have four suggestions for future research. First, studies involving long COVID should state the definitions they use. We do not recommend that authors use their own definitions, because this will make it more difficult to summarize the evidence. It is better to use the definitions published by the NICE or WHO. Second, to strengthen the reliability of our results, more studies exploring the effect of vaccines on long COVID are needed in the future. In addition to whether long COVID has occurred, studies should focus on the development of long COVID symptoms. Third, it is important to explore how vaccines can prevent long COVID in basic research. Basic studies have a great reference value for examining the current doubts about mechanisms. Fourth, a large number of people have been vaccinated with inactivated vaccines, and there is an urgent need to assess the effect of inactivated vaccines on long COVID.

This study has two advantages. First, to the best of our knowledge, this is the first systematic review with a meta-analysis to quantifiably assess the effect of COVID-19 vaccines on long COVID. The protective effect of vaccination against long COVID has been found. This study provides evidence-based medical information on this topic. Second, the primary meta-analysis was statistically significant in the sensitivity analysis. The stability of the result was good. This study has four limitations. First, the heterogeneity of the studies was high, which had an impact on the reliability of the results. More studies are needed in the future to calculate the effect values. Second, the definitions of long COVID are different among the included studies (especially observation time), although the NICE and WHO have both defined long COVID. This problem has also been considered in other studies [50,51]. It may be a source of heterogeneity in our study. Third, the data on some long COVID symptoms were insufficient to perform meta-analyses. For long COVID symptoms with meta-analyses, there were few pooled studies. The concept of fatigue associated with this syndrome is often also underestimated. Fourth, most of the participants were vaccinated with mRNA, and data on inactivated vaccines were lacking.

## 5. Conclusions

COVID-19 vaccines were found to have an effect on reducing the risk of long COVID. The protective effect was found in participants vaccinated with two doses, but not one dose. Regardless of whether being vaccinated before or after SARS-CoV-2 infection/COVID, vaccination was effective against long COVID. We suggest that the vaccination rate should be improved as soon as possible, especially for a complete vaccination course. It is better to be vaccinated so as to reduce the risk of long COVID, regardless of whether or not a patient has been infected with SARS-CoV-2. There should be more studies done to explore the basic mechanism of the protective effect of COVID-19 vaccines on long COVID in the future.

## Figures and Tables

**Table 1:** Characteristics of the included studies.

Study ID	Study Design	Nationality of Population	Age (Mean ± SD or Range) (Years)	Vaccination Time	Type of Vaccine	Definition of Long COVID *	Sample Size for Meta-Analysis	Quality Assessment
Nehme 2022 [21]	Cross-sectional study	Switzerland	43.5 ± 13.7	After SARS-CoV-2 infection	mRNA-1273, BNT162b2	Presence of fatigue, difficulty concentrating or memory loss, loss of or change in smell, loss of or change in taste, shortness of breath, and headache more than 6 months after an infection	1596	Low risk
Ayoubkhani 2022 [7]	Cohort study	UK	18–69	Before SARS-CoV-2 infection	ChAdOx1 nCoV-19, BNT162b2, mRNA-1273	Presence of symptoms more than 4 weeks after the first having COVID-19, that are not explained by something else	6180	Moderate risk
Kuodi 2022 [32]	Cross-sectional study	Israel	≥19	Before and after SARS-CoV-2 infection	Mainly BNT162b2	No clear definition	951	Low risk
Alghamdi 2022 [22]	Cross-sectional study	Saudi Arabia	12–70	NA	ChAdOx1 nCoV-19, BNT162b2	No clear definition	2218	Moderate risk
Simon 2021 [34]	Cohort study	USA	NA	Before and after COVID-19 diagnosis	NA	Presence of one or more COVID-associated symptoms between 12 and 20 weeks after the initial COVID-19 diagnosis	240,648	Low risk
Taquet 2022 [24]	Cohort study	USA	57.0 ± 17.9	Before SARS-CoV-2 infection	BNT162b2, mRNA-1273, Ad26.COV2.S, other COVID-19 vaccines	Presence of chest/throat pain, abnormal breathing, abdominal symptoms, fatigue/malaise, anxiety/depression, pain, headache, cognitive dysfunction, and myalgia between 90 and 120 days after COVID-19 diagnosis	9953	Low risk
Otmani 2022 [23]	Case-control study	Morocco	NA	After contracting the COVID-19 infection	NA	Guideline published by the NICE	118	Low risk
Azzolini 2022 [15]	Cohort study	Italy	44.3 ± 10.7 (with long COVID); 41.2 ± 11.4 (without long COVID)	Before SARS-CoCV-2 infection	BNT162b2	Prescence at least 1 SARS-CoV-2-related symptom with a duration of more than 4 weeks	739	Moderate risk
Wynberg 2022 [16]	Cohort study	Netherlands	53.5 (IQR: 41.0–64.0)	After SARS-CoV-2 infection	BNT162b2, mRNA-1273, ChAdOx1 nCoV-19, Ad26.COV2.S	Criteria published by the WHO	315	Low risk
Al-Aly 2022 [33]	Cohort study	USA	66.63 ± 13.84	Before SARS-CoV-2 infection	BNT162b2, mRNA-1273, Ad26.COV2.S	The symptoms starting from 30 days after the first positive SARS-CoV-2 test	147,414	Low risk
Fernández 2022 [25]	Cohort study	Spain	41.0 ± 16.8	Before or after COVID-19 diagnosis	BNT162b2, mRNA-1273, ChAdOx1 nCoV-19, Ad26.COV2.S	Prescence of symptoms that persisted for more than 3 weeks after the initial infection and cannot be explained by other causes	110,726	Low risk
Messiah 2022 [26]	Cohort study	USA	5–19	NA	NA	Guideline published by the NICE	1748	Low risk
Meza-Torres 2022 [27]	Cohort study	UK	44.5 ± 21.77	Before or after COVID-19 diagnosis	NA	Presence of fatigue, breathlessness, cognitive dysfunction, and a variety of other symptoms occurring more than 28 days after COVID-19 infection	408,882	Low risk
Peghin 2022 [28]	Cohort study	Italy	≥18	After COVID-19 diagnosis	BNT162b2, mRNA-1273, ChAdOx1 nCoV-19, Ad26.COV2.S	Guideline published by the NICE	479	Low risk
Pinato 2022 [29]	Cohort study	UK, Italy, Spain	≥18	Before SARS-CoV-2 infection	BNT162b2, mRNA-1273, ChAdOx1 nCoV-19, Ad26.COV2.S	Presence of long-term effects start at least 4 weeks after infection	1228	Low risk
Zisis 2022 [30]	Cohort study	USA	≥18	After COVID-19 diagnosis	NA	Prescence of new, continuing, or recurrent symptoms that occur 4 or more weeks after the initial SARS-CoV-2 infection	50,450	Low risk
Budhiraja 2022 [35]	Cross-sectional study	India	<18-≥75	Before COVID-19 diagnosis	ChAdOx1nCoV-19, a whole-virion inactivated vero cell derived vaccine (available as Covaxin in India)	Presence of any symptoms after discharge from the hospital	5529	Low risk
Hajjaji 2022 [31]	Cross-sectional study	France	≥18	NA	NA	Persistent symptoms of SARS-CoV-2 infection lasting more than 6 months	168	Moderate risk

* Definition published by NICE: the term “long COVID” is commonly used to describe signs and symptoms that continue or develop after acute COVID-19. It includes both ongoing symptomatic COVID-19 (from 4 to 12 weeks) and post-COVID-19 syndrome (12 weeks or more) [36].

**Table 2:** Results of the subgroup analysis of the effect of vaccination on long COVID.

Subgroups	The Number of Studies	The Number of People	I^2^ (%)	RR (95% CI)	*p* Value of Meta-analysis
The number of vaccine doses					
1 dose	6	655,962	99	0.83 (0.65–1.07)	0.14
2 doses	7	420,402	90	0.83 (0.74–0.94)	<0.01
Age					
<60 years	3	12,415	89	0.76 (0.54–1.06)	0.11
≥60 years	2	9509	55	0.87 (0.60–1.24)	0.43
Vaccination time					
Before SARS-CoV-2 infection/COVID-19	6	180,996	97	0.82 (0.74–0.91)	<0.01
After SARS-CoV-2 infection/COVID-19	4	2508	24	0.83 (0.74–0.92)	<0.01
Definition of long COVID					
Presence of symptoms more than 4 weeks after SARS-CoV-2 infection/COVID-19 diagnisis *	7	419,374	87	0.68 (0.53–0.87)	<0.01
Other definitions	8	526,302	99	0.75 (0.64–0.88)	<0.01

* This subgroup contained 3 studies that used the NICE definition.

**Table 3:** Effects of vaccination on long COVID symptoms.

Long COVID Symptom	The Number of Studies	The number of People	I^2^ (%)	RR (95% CI)	*p* Value of Meta-Analysis
Anxiety and/or depression	4	28,604	70	0.83 (0.67–1.03)	0.08
Chest or throat pain	3	26,386	0	1.01 (0.95–1.08)	0.67
Cognitive dysfunction/symptoms	2	22,124	8	0.89 (0.83–0.96)	<0.01
Fatigue	6	225,478	97	0.77 (0.58–1.02)	0.07
Hair loss	2	6480	50	0.86 (0.62–1.19)	0.37
Headache/migraine	4	76,836	99	0.95 (0.50–1.79)	0.87
Kidney diseases/problems	2	148,365	0	0.68 (0.64–0.73)	<0.01
Loss of concentration	2	6480	71	0.65 (0.35–1.19)	0.16
Loss of smell	3	8698	75	0.67 (0.36–1.26)	0.21
Loss of taste	3	8698	68	0.71 (0.48–1.07)	0.10
Myalgia	2	25,435	15	0.68 (0.62–0.74)	<0.01
Nausea and/or vomiting	2	6480	87	0.80 (0.31–2.02)	0.63
Respiratory symptoms/sequelae	5	78,064	98	0.91 (0.60–1.40)	0.68
Sleeping disorders/problem sleeping	3	8698	25	0.74 (0.64–0.86)	<0.01
Weight loss	2	6480	95	1.24 (0.22–7.05)	0.81

## Data Availability

Data are available from the corresponding author upon request.

## Supplementary Materials

The following supporting information can be downloaded at: https://www.mdpi.com/article/10.3390/ijerph191912422/s1, Table S1: Search strategy. Table S2: Data from the meta-analysis. Table S3: Quality assessment of the studies. Table S4: Sensitivity analysis by excluding one study every time.
