# Supplementary material for: Effect of COVID-19 Vaccines on Reducing the Risk of Long COVID in the Real World: A Systematic Review and Meta-Analysis
Source: Int J Environ Res Public Health. 2022 Sep 29;19(19):12422. doi: 10.3390/ijerph191912422 (PMC9566528; doi:10.3390/ijerph191912422)
Supplement: Supplementary file 1 [file ijerph-19-12422-s001.zip › ijerph-1928937-supplementary.pdf]

Table S1. Search strategy.

Database	Search query
PubMed	((COVID-19 Vaccines[MeSH Terms]) OR (Vaccines[MeSH Terms]) OR (Vaccination[MeSH Terms]) OR (Vaccin*[Title/Abstract])) AND ((post-acute COVID-19 syndrome[Supplementary Concept]) OR (post-acute COVID-19 syndrome[Title/Abstract]) OR (long-COVID[Title/Abstract]) OR (long-haul COVID[Title/Abstract]) OR (post-acute COVID syndrome[Title/Abstract]) OR (persistent COVID-19[Title/Abstract]) OR (long hauler COVID[Title/Abstract]) OR (long COVID[Title/Abstract]) OR (post-acute sequelae of SARS-CoV-2 infection[Title/Abstract]) OR (long haul COVID[Title/Abstract]) OR (chronic COVID syndrome[Title/Abstract]) OR (post COVID-19[Title/Abstract]) OR (post-acute COVID-19[Title/Abstract]) OR (COVID-19 sequelae[Title/Abstract]) OR (post-acute sequelae of COVID-19[Title/Abstract]) OR (post COVID-19 condition[Title/Abstract]) OR (long-term symptoms[Title/Abstract]))
Embase	(‘sars-cov-2 vaccine’/exp OR ‘vaccine’/exp OR ‘vaccin*’:ab,ti) AND (‘long COVID’/exp OR (‘long COVID’ OR ‘chronic COVID syndrome’ OR ‘chronic COVID-19’ OR ‘COVID long-hauler’ OR ‘COVID-19 long-hauler’ OR ‘long haul COVID’ OR ‘long hauler COVID’ OR ‘post COVID 19’ OR ‘post-acute COVID’ OR ‘post COVID-19’ OR ‘post-acute COVID-19’ OR ‘COVID-19 sequelae’ OR ‘post-acute sequelae of COVID-19’ OR ‘post COVID-19 condition’ OR ‘long-term symptoms’):ab,ti)

Web of science	(TS= ("COVID-19 Vaccines" OR "sars-cov-2 vaccine" OR "vaccine" OR "vaccination") OR TI= ("vaccin*") OR AB= ("vaccin*")) AND (TS= ("post-acute COVID-19 syndrome" OR "long COVID") OR TI= ("post-acute COVID-19 syndrome" OR "long COVID" OR "long-haul COVID" OR "post-acute COVID syndrome" OR "persistent COVID-19" OR "post-acute sequelae of SARS-CoV-2 infection" OR "chronic COVID syndrome" OR "chronic COVID-19" OR "COVID long-hauler" OR "COVID-19 long-hauler" OR "long haul COVID" OR "long hauler COVID" OR "post COVID 19" OR "post-acute COVID" OR "post COVID-19" OR "post-acute COVID-19" OR "COVID-19 sequelae" OR "post-acute sequelae of COVID-19" OR "post COVID-19 condition" OR "long-term symptoms") OR AB= ("post-acute COVID-19 syndrome" OR "long COVID" OR "long-haul COVID" OR "post-acute COVID syndrome" OR "persistent COVID-19" OR "post-acute sequelae of SARS-CoV-2 infection" OR "chronic COVID syndrome" OR "chronic COVID-19" OR "COVID long-hauler" OR "COVID-19 long-hauler" OR "long haul COVID" OR "long hauler COVID" OR "post COVID 19" OR "post-acute COVID" OR "post COVID-19" OR "post-acute COVID-19" OR "COVID-19 sequelae" OR "post-acute sequelae of COVID-19" OR "post COVID-19 condition" OR "long-term symptoms"))
ScienceDirect	The following search query was used for the title, abstract, and author-specified keywords of article. Due to the limitation on the number of terms in the ScienceDirect database, the search query was divided into four parts.

Part1: ((COVID-19 Vaccine) OR (vaccine) OR (vaccination)) AND ((post-acute COVID-19 syndrome) OR (long-COVID) OR (long-haul COVID) OR (persistent COVID-19) OR (long hauler COVID))
Part2: ((COVID-19 Vaccine) OR (vaccine) OR (vaccination)) AND ((long COVID) OR (post-acute sequelae of SARS-CoV-2 infection) OR (long haul COVID) OR (chronic COVID syndrome) OR (chronic COVID-19))
Part3: ((COVID-19 Vaccine) OR (vaccine) OR (vaccination)) AND ((COVID long-hauler) OR (COVID-19 long-hauler) OR (post COVID 19) OR (post-acute COVID) OR (post COVID-19))
Part4: ((COVID-19 Vaccine) OR (vaccine) OR (vaccination)) AND ((post-acute COVID-19) OR (COVID-19 sequelae) OR (post-acute sequelae of COVID-19) OR (post COVID-19 condition) OR (long-term symptoms))

Table S2. Data for meta-analysis.

Study ID	Age (mean±SD or range) (years)	The number of doses	Sample size for meta-analysis	At least one symptom	Anxiety and/or depression	Chest or throat pain	Cognitive dysfunction/ symptoms	Fatigue	Hair loss	Headache/ migraine	Kidney diseases/problems	Loss of concentration	Loss of smell	Loss of taste	Myalgia	Nausea and/or vomiting	Respiratory symptoms/sequelae	Sleeping disorders/problem sleeping	Weight loss
Nehme 2022	43.5±13.7	2	347	69															
		1	424	102															
		0	825	241															
	<40	2	93	19															
		1	138	31															
		0	473	138															
	40-59	2	166	40															
		1	229	53															
		0	302	88															
	≥60	2	88	10															
		1	57	18															
		0	50	15															
Ayoubkhani 2022	18-69	2	3090	294															
		0	3090	452															
Kuodi 2022	44	2	294		17	14		33	9	41	2	13	9	15		7	14	14	8
	52	1	340		19	24		93	43	80	5	44	9	20		11	29	42	6
	39	0	317		19	23		82	36	69	5	33	23	28		19	25	29	14
Alghamdi 2022	12-70	2	1696		497		471						1048	1048				406	
		1	360		129		100						250	250				87	
		0	162		60		44						114	114				48	

Simon 2021	NA	1	20,188	7532								
		0	220,460	123,365								
Taquet 2022	57.0±17.9	2	6957	4518	2159	1163	831	1444	1122		443	1698
		0	6957	4566	2245	1160	901	1414	1087		645	1759
		1	2996	1927	910	456	327	548	477		162	729
		0	2996	1985	956	442	408	567	463		221	726
	<60	≥1	4633	2893	1505	680	242	718	840		206	959
		0	4633	2868	1344	661	287	601	823		259	947
	>60	≥1	4657	3129	1544	865	815	1099	676		409	1344
		0	4657	3231	1432	857	827	1146	723		442	1383
Otmani 2022	NA	≥1	63	31								
		0	55	25								
Azzolini 2022	44.3±10.7 (with long COVID); 41.2±11.4 (without long COVID)	3	262	42								
		2	46	8								
		1	10	3								
		0	421	176								
Wynberg 2022	53.5 (IQR: 41.0-64.0)	≥1	216	119								
		0	99	66								
Al-Aly 2022	66.63±13.84	≥1	33,940	10,842				822		949		
	57.81±15.94	0	113,474	41,173				3808		4644		
Fernández 2022	41.0±16.8	⩾1	98,715	394								
		0	12,011	161								
Messiah 2022	5-19	Fully vaccinated	475	13								

		Partially vaccinated	142	1													
		0	1131	67													
Meza-Torres 2022	44.5±21.77	2	726	10													
		1	15,832	266													
		0	392,324	7347													
Peghin 2022	⩾18	⩾1	132	59													
		0	347	167													
Pinato 2022	⩾18	Fully vaccinated	60	5			4										
		Partially vaccinated	33	4			2										
		0	1135	195			70										
Zisis 2022	⩾18	Fully vaccinated	25,225				605		247								
		0	25,225				1268		635								
Budhiraja 2022	<18-⩾75	2	913	718	28	73	143	14	90	16	4	10	89	63	53	99	50
		0	4616	3571	260	341	795	59	194	172	25	98	732	259	135	736	87
Hajjaji 2022	⩾18	2	44	7													
		1	78	19													
		0	46	11													

Table S3. Quality assessment of included studies.

NOS for cohort study										
	Selection				Comparability	Outcome				
Study ID	Representativeness of the exposed cohort	Selection of the non exposed cohort	Ascertainment of exposure	Demonstration that outcome of interest was not present at start of study	Comparability of cohorts on the basis of the design or analysis	Assessment of outcome	Was follow-up long enough for outcomes to occur	Adequacy of follow up of cohorts	Total scores	Risk of bias
Ayoubkhani 2022	1	1	0	0	1	0	1	1	5	Moderate risk
Simon 2021	1	1	1	0	2	1	1	1	8	Low risk
Taquet 2022	1	1	1	0	2	1	1	1	8	Low risk
Azzolini 2022	0	1	0	0	2	0	1	1	5	Moderate risk
Wynberg 2022	1	1	1	1	2	0	1	1	8	Low risk
Al-Aly 2022	1	1	1	0	2	1	1	1	8	Low risk
Fernández 2022	1	1	1	0	2	1	1	1	8	Low risk
Messiah 2022	0	1	1	1	2	0	1	1	7	Low risk
Meza-Torres 2022	1	1	1	0	2	1	1	1	8	Low risk
Peghin 2022	0	1	1	1	2	1	1	1	8	Low risk
Pinato 2022	1	1	1	0	2	1	0	1	7	Low risk
Zisis 2022	1	1	1	0	2	1	1	1	8	Low risk

NOS for case-control study				
Study ID	Selection	Comparability	Exposure	Risk of bias

	Is the case definition adequate	Representativeness of the cases	Selection of Controls	Definition of Controls	Comparability of cases and controls on the basis of the design or analysis	Ascertainment of exposure	Same method of ascertainment for cases and controls	Non-Response rate	Total scores	
Otmani 2022	1	1	1	1	2	0	1	0	7	Low risk

AHRQ for cross-sectional study												
Study ID	Items										Total scores	Risk of bias
	Define the source of information (survey, record review)	List inclusion and exclusion criteria for exposed and unexposed subjects (cases and controls) or refer to previous publications	Indicate time period used for identifying patients	Indicate whether or not subjects were consecutive if not population-based	Indicate if evaluators of subjective components of study were masked to other aspects of the status of the participants	Describe any assessments undertaken for quality assurance purposes	Explain any patient exclusions from analysis	Describe how confounding was assessed and/or controlled	If applicable, explain how missing data were handled in the analysis	Summarize patient response rates and completeness of data collection	Clarify what follow-up, if any, was expected and the percentage of patients for which incomplete data or follow-up was obtained		
Nehme 2022	1	1	1	1	1	0	0	1	0	1	1	8	Low risk
Kuodi 2022	1	1	1	1	1	1	1	1	0	1	1	10	Low risk
Alghamdi 2022	1	1	0	1	1	0	0	1	0	0	1	6	Moderate risk
Budhiraja 2022	1	1	1	1	1	0	1	1	0	1	0	8	Low risk
Hajjaji 2022	1	1	1	1	1	0	0	0	0	1	1	7	Moderate risk

Table S4. Sensitivity analysis by excluding a study every time.

Excluded study	I^2 (%)	RR (95% CI)	P value of meta-analysis
Aly-Aly 2022	99	0.70 (0.57-0.87)	<0.01
Ayoubkhani 2022	99	0.72 (0.58-0.89)	<0.01
Azzolini 2022	99	0.74 (0.61-0.90)	<0.01
Budhiraja 2022	99	0.69 (0.56-0.85)	<0.01
Fernández 2022	99	0.77 (0.67-0.90)	<0.01
Hajjaji 2022	99	0.71 (0.57-0.87)	<0.01
Messiah 2022	99	0.74 (0.60-0.90)	<0.01
Meza-Torres 2022	99	0.70 (0.57-0.86)	<0.01
Nehme 2022	99	0.71 (0.57-0.88)	<0.01
Otimani 2022	99	0.69 (0.57-0.85)	<0.01
Peghin 2022	99	0.70 (0.57-0.86)	<0.01
Pinato 2022	99	0.72 (0.59-0.89)	<0.01
Simon 2021	96	0.72 (058-0.89)	<0.01
Taquet 2022	99	0.69 (0.56-0.85)	<0.01
Wynberg 2022	99	0.70 (0.57-0.87)	<0.01
